# Anti-cancer efficacy of SREBP inhibitor, alone or in combination with docetaxel, in prostate cancer harboring p53 mutations

**DOI:** 10.18632/oncotarget.5879

**Published:** 2015-10-16

**Authors:** Xiangyan Li, Jason Boyang Wu, Leland W.K. Chung, Wen-Chin Huang

**Affiliations:** ^1^ Uro-Oncology Research Program, Department of Medicine, Samuel Oschin Comprehensive Cancer Institute, Cedars-Sinai Medical Center, Los Angeles, CA 90048, USA

**Keywords:** mutant p53, SREBP, prostate cancer, docetaxel

## Abstract

Mutant p53 proteins (mutant p53s) have oncogenic gain-of-function properties correlated with tumor grade, castration resistance, and prostate cancer (PCa) tumor recurrence. Docetaxel is a standard first-line treatment for metastatic castration-resistant PCa (mCRPC) after the failure of hormone therapy. However, most mCRPC patients who receive docetaxel experience only transient benefits and rapidly develop incurable drug resistance, which is closely correlated with the p53 mutation status. Mutant p53s were recently reported to regulate the metabolic pathways via sterol regulatory element-binding proteins (SREBPs). Therefore, targeting the SREBP metabolic pathways with docetaxel as a combination therapy may offer a potential strategy to improve anti-tumor efficacy and delay cellular drug resistance in mCRPC harboring mutant p53s. Our previous data showed that fatostatin, a new SREBP inhibitor, inhibited cell proliferation and induced apoptosis in androgen receptor (AR)-positive PCa cell lines and xenograft mouse models. In this study, we demonstrated that mutant p53s activate the SREBP-mediated metabolic pathways in metastatic AR-negative PCa cells carrying mutant p53s. By blocking the SREBP pathways, fatostatin inhibited cell growth and induced apoptosis in metastatic AR-negative PCa cells harboring mutant p53s. Furthermore, the combination of fatostatin and docetaxel resulted in greater proliferation inhibition and apoptosis induction compared with single agent treatment in PCa cells *in vitro* and *in vivo*, especially those with mutant p53s. These data suggest for the first time that fatostatin alone or in combination with docetaxel could be exploited as a novel and promising therapy for metastatic PCa harboring p53 mutations.

## INTRODUCTION

The tumor suppressor TP53 gene, which resides on chromosome 17p13.1 and encodes the p53 protein, is one of the most frequent targets for mutation in human cancers [1, 2]. Mutant p53 proteins (mutant p53s) are incapable of recognizing wild-type p53 (wtp53) DNA binding sites. Some mutations acquire new and distinct oncogenic properties, which are generally referred to as “gain of function” (GOF) effects [3]. TP53 mutations have been reported in 3%–20% of prostate cancers (PCa) [4–6], and are often correlated with tumor grade, castration resistance, and tumor recurrence [5, 6]. These properties make mutant p53 an extremely attractive target for PCa therapy.

Docetaxel is a taxane agent that has been widely used as the front-line treatment for metastatic castration-resistant PCa (mCRPC) after the failure of androgen deprivation therapy [7]. However, most mCRPC patients who receive docetaxel experience only transient benefit and rapidly develop incurable drug resistance. Recent evidence demonstrated that mutant p53 plays a critical role in the development of docetaxel resistance in PCa patients [8]. There is a compelling need to discover new approaches targeting mutant p53s to treat lethal PCa progression.

Mutant p53s were recently reported to regulate metabolic pathways via regulation of transcription factors, sterol regulatory element-binding proteins (SREBPs), in breast cancer [9, 10]. SREBPs are basic helix-loop-helix leucine (bHLH) zipper transcription factors that transcriptionally activate genes involved in fatty acid and cholesterol biosynthesis and homeostasis [11, 12]. Previous studies revealed that SREBPs were highly elevated in PCa specimens with aggressive pathologic features and also promoted PCa cell growth and lethal progression [13, 14]. Targeting the SREBP metabolic pathways in a docetaxel-based combination therapy may offer a potential strategy to improve anti-tumor efficacy and overcome cellular drug resistance for the treatment of mCRPC harboring mutant p53s.

SREBPs are the potential targets for the treatment of androgen receptor (AR)-positive PCa [15, 16]. However, the role of SREBPs in AR-negative PCa and PCa with p53 mutations has been unexplored. In this study, we revealed for the first time that mutant p53s activate the SREBP-mediated metabolic pathways in AR-negative PCa cells. Furthermore, treatment with a SREBP inhibitor, fatostatin [16–18], inhibited cell growth and induced caspase-dependent apoptosis in metastatic AR-negative PCa cells, especially those harboring mutant p53s. In addition, *in vitro* and *in vivo* studies demonstrated that the combination of fatostatin and docetaxel resulted in greater anti-tumor activity compared to single agent in PCa harboring mutant p53s. These data suggest that fatostatin alone or in combination with docetaxel could be exploited as a novel and promising therapy for aggressive PCa bearing p53 mutations.

## RESULTS

### Mutant p53 proteins activate the SREBP-mediated signaling pathways in metastatic PCa cells

**Figure 1:**
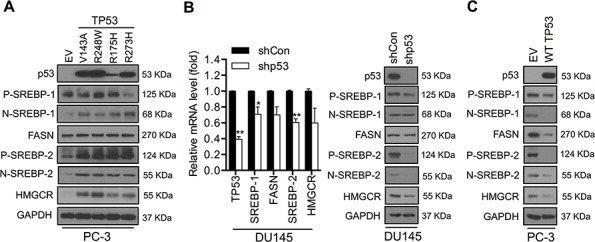
Mutant p53 activates the SREBP-mediated signaling pathways in PCa cells **A.** Western blot analysis of p53, SREBP-1, SREBP-2, FASN and HMGCR expression in PC-3 cells stably transfected with various mutant p53s (V143A, R248W, R175H and R273H) or empty vector (EV). GAPDH was used as a loading control. *P* and *N* denote the precursor and nuclear forms of SREBPs, respectively. **B.** qPCR (left panel) and Western blot (right panel) analyses of p53, SREBP-1, SREBP-2, FASN and HMCCR expression in DU145 cells infected with p53-targeting shRNAs (shp53) or scrambled shRNA (shCon) lentivirus particles. qPCR data were normalized to β-actin and represent the mean ± SD of three independent triplicate experiments. *P < 0.05, **P < 0.01. GAPDH was used as a loading control for Western blot analysis. **C.** Western blot analysis of p53, SREBP-1, SREBP-2, FASN and HMCCR expression in PC-3 cells transiently transfected with wild-type p53 plasmid (WT TP53) or empty vector (EV). GAPDH was used as a loading control. *P* and *N* denote the precursor and nuclear forms of SREBPs, respectively.

To investigate the frequency of p53 mutations in PCa, we analyzed the codon distribution of p53 mutations using the IARC TP53 Mutation Database (R17, November 2013). TP53 mutations in PCa typically occur within the DNA-binding domain (amino acid 102–292) with hot spots at codons R175, G245, R248 and R273 (Supplementary Figure 1A). Mutations are subdivided into “contact mutations” that eliminate an essential DNA contact (e.g., R273H and R248W) or “structural mutations” that result in structural perturbations (R175H, V143A, G245S, Y220C, R249S, and R282W) [19, 20]. To further study the functional impact of TP53 mutations on the SREBP-mediated metabolic pathways, we selected human metastatic PCa cell lines with various TP53 status, such as PC-3 (p53-null) and DU145 (heterozygous p53, P223L and V274F), as cell models. First, we established stable PC-3 cell clones expressing various mutant p53s, including V143A, R248W, R175H or R273H. PC-3 cells transfected with an empty vector (EV) were developed as a control (Figure 1A). Overexpression of mutant p53s showed no effects on cell growth in PC-3 cells. Only R248W mutation significantly increased cell growth compared with control cells after 6-day incubation (Supplementary Figure 1B).

It has been reported that mutant p53s are recruited to the promoters of genes encoding mevalonate pathway enzymes via SREBP proteins [9]. This is consistent with our observation that expression of SREBP-1, SREBP-2 and their downstream target proteins, including fatty acid synthase (FASN) and HMG-CoA reductase (HMGCR), was increased in mutant p53s overexpressing PC-3 cells (Figure 1A). Next, we evaluated whether the lack of p53 expression causes inhibition of the SREBP-mediated pathways in metastatic PCa cells. Lentivirus-mediated p53 short hairpin RNA (shp53) interference was used to knock down endogenous expression of mutant p53s in DU145 cells. Suppression of endogenous mutant p53s expression resulted in a significant decrease of SREBP-2 and HMGCR expression, with slight decreases of SREBP-1 and FASN expression at the mRNA and protein levels in DU145 cells (Figure 1B). Moreover, downregulation of mutant p53s led to reduced cell growth and colony formation compared with controls (shCon) in DU145 cells (Supplementary Figure 1C). In addition, transient expression of wild-type p53 (wtp53) in PC-3 cells was sufficient to down-regulate the expression of SREBPs and their target genes (Figure 1C). Collectively, our data suggest that mutant p53s significantly activate the SREBP-mediated metabolic pathways in metastatic PCa cells.

### Fatostatin inhibits cell proliferation and colony formation in PCa cells harboring different p53 status

Mutant p53 proteins gain oncogenic properties that enable them to promote cancer cell invasion, metastasis and survival, making them extremely attractive targets for PCa treatment [21]. Current strategies have focused on destabilization or inactivation of mutant p53s [22], reactivation of wild-type function in the mutant p53 protein [23] or targeting the downstream pathways mediated by mutant p53s such as transforming growth factor (TGF-β) receptor, epithelial growth factor receptor (EGFR) and MET (the receptor for hepatocyte growth factor, HGF) [24–26]. Fatostatin was recently developed and identified as a SREBP inhibitor that perturbs the nuclear translocation of SREBPs and inhibits their transcriptional activity. Our previous study showed that fatostatin inhibited cell growth and induced apoptosis by blocking the SREBP-regulated metabolic pathways and androgen receptor (AR) signaling in AR-positive PCa cells. However, the effect of fatostatin on metastatic PCa cells harboring null or mutant p53s is unknown.

**Figure 2:**
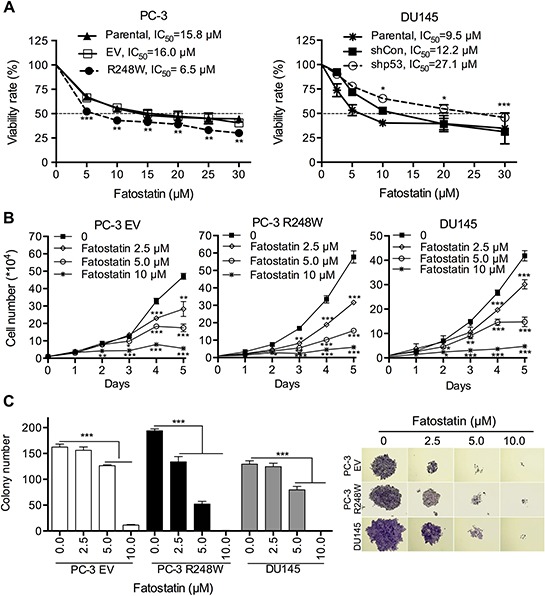
Fatostatin inhibits cell proliferation and colony formation in PCa cells harboring different p53 status **A.** Fatostatin suppressed the proliferation of PCa cells of different p53 status. Cells (PC-3 Parental, EV, R248W; DU145 Parental, shCon, shp53) were treated with various concentrations of fatostain for 72 hours and the IC_50_ values were calculated and shown from three independent experiments. *P < 0.05, **P < 0.01 and ***P < 0.001. **B.** Fatostatin inhibited growth of PC-3 EV, PC-3 R248W and DU145 cells in a time- and dose-dependent manner. Cell growth was determined by counting cell numbers daily using a hemocytometer. *P < 0.05, **P < 0.01 and ***P < 0.001. **C.** Fatostatin decreased colony formation of PC-3 EV, PC-3 R248W and DU145 cells in a dose-dependent manner after 10-day treatment. The number of colonies was counted and reported as the mean ± SD of triplicate experiments. ***P < 0.001. Colony development images are shown in the right panel.

Established PCa cell clones with different p53 status were treated with various concentrations of fatostatin for 72 hours and cell viability was determined. The half maximal inhibitory concentrations (IC_50_) of fatostatin in different PC-3 cell clones ranged from 6–20 μM (Figure 2A, left panel and Supplementary Figure 2A). Of note, fatostatin effectively inhibited the growth of PC-3 R248W (IC_50_ = 6.5 μM), which exhibited higher mutant p53-activated SREBP signaling compared with other cell clones, suggesting that PC-3 R248W cells were more sensitive to fatostatin. For DU145 cells carrying endogenous mutant p53s, the concentration of fatostatin that inhibited 50% of cell proliferation was 9.5 μM, suggesting that the sensitivity of p53 mutant cells to fatostatin was higher than that of null p53 cells (PC-3 parental cells, IC_50_ = 15.8 μM; and PC-3 EV cells, IC_50_ = 16.0 μM). However, the IC_50_ value of fatostatin in p53-silenced DU145 cells was 2-fold higher than that in the control cell clone (shCon) (Figure 2A, right panel). These data suggest that p53 mutation was a determinant of fatostatin sensitivity in PCa cells.

Based on the prevalence of contact mutation and the sensitivity to fatostatin, PC-3 R248W and DU145 cells harboring mutant p53s were excellent cell models for subsequent experiments. We next determined the effects of fatostatin on cell growth and colony formation in PC-3 EV, PC-3 R248W and DU145 cells. Fatostatin led to reduction in cell growth in a dose- and time-dependent manner in these PCa cell clones (Figure 2B). Notably, significant inhibition of PC-3 R248W and DU145 cell growth by fatostatin at 5.0 μM occurred early (day 2), indicating that fatostatin showed stronger anti-growth effect on p53 mutant cells than p53 null cells. In addition, fatostatin inhibited the number and size of colony development in a dose-dependent manner in PC-3 EV, PC-3 R248W and DU145 cells (Figure 2C). We also observed that a lower concentration of fatostatin (2.5 μM) displayed significant inhibition on the number of colony formation in PC-3 R248W cells. Collectively, these data suggest that fatostatin suppresses cell proliferation and clonogenicity in PCa cells, especially those carrying mutant p53s.

### Fatostatin causes G_2_/M cell cycle arrest and induces apoptosis in PCa cells harboring mutant p53 status

**Figure 3:**
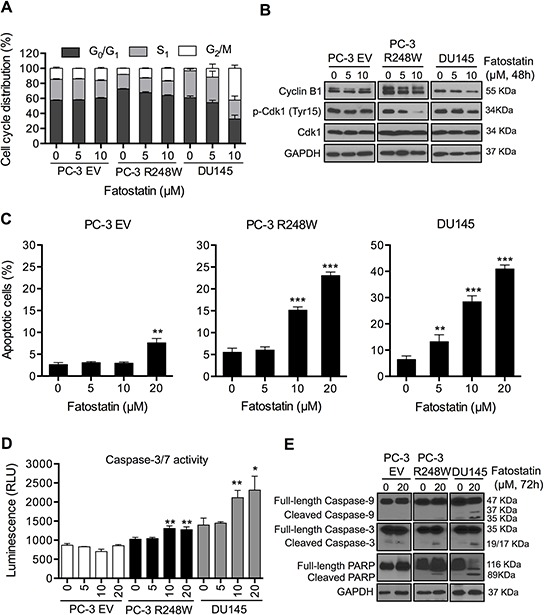
Fatostatin causes G_2_/M cell cycle arrest and induces apoptosis in PCa cells harboring p53 mutations **A.** Fatostatin caused G_2_/M cell cycle arrest after 48-hour treatment compared with vehicle in PCa cells harboring p53 mutations. Cell cycle distributions of PC-3 EV, PC-3 R248W and DU145 cells treated with fatostatin or vehicle were assessed by flow cytometry. Data represent the means ± SD values from triplicate experiments. **B.** Fatostatin decreased the expression of Cyclin B1 and p-Cdk1 (Tyr15) in PC-3 R248W and DU145 cells but not in PC-3 EV cells as determined by Western blot. **C.** Apoptosis in PC-3 EV, PC-3 R248W and DU145 cells treated by fatostatin was determined by flow cytometry-based Annexin V-FITC and PI staining analysis. **P < 0.01, ***P < 0.001. **D.** Caspase-3/7 activity in PC-3 EV, PC-3 R248W and DU145 cells treated with vehicle or fatostatin for 48 hours was determined by an enzymatic activity assay. Data are plotted as the relative units of luciferase intensity and reported as the means ± SD values from triplicate experiments. *P < 0.05, **P < 0.01. **E.** Western blot analysis of apoptosis-related markers (caspase-9, caspase-3 and PARP) in PC-3 EV, PC-3 R248W and DU145 cells treated with 20 μM of fatostatin for 72 hours. GAPDH was used as a loading control.

Given the growth inhibitory effect of fatostatin in PCa cells, we next investigated the potential mechanisms underlying this inhibition. To determine the impact of fatostatin on cell cycle distribution, PC-3 EV, PC-3 R248W and DU145 cells were treated with fatostatin for 48 hours, stained with propidium iodide (PI) and then analyzed by flow cytometry. As shown in Figure 3A, treatment with fatostatin resulted in an increase of the percentage of cells in the G_2_/M phase in PC-3 R248W and DU145 cells but not in PC-3 EV cells compared with vehicle-treated cells, respectively. Next, we assessed the effects of fatostatin on G_2_/M cell cycle-related proteins, including Cyclin B1, Cdk1 and p-Cdk1 (Tyr15). Western blot results demonstrated that fatostatin inhibited expression of Cyclin B1 and p-Cdk1 in a dose-dependent pattern in PC-3 R248W and DU145 cells (Figure 3B). To explore whether fatostatin induced apoptosis, flow cytometry-based Annexin V-FITC/PI staining, caspase enzymatic activity assay and Western blot analysis of apoptosis-related proteins were performed. Annexin V-FITC/PI results showed that fatostatin induced only slight apoptotic cell death at 20 μM in PC-3 EV cells after 72-hour treatment (Figure 3C, left panel), while PC-3 R248W and DU145 cells treated with fatostatin underwent a large increase in the percentage of apoptotic cells (Figure 3C, middle and right panels). Subsequently, caspase-3/7 activity was examined in PC-3 EV, PC-3 R248W and DU145 cells after fatostatin treatment for 48 hours. As shown in Figure 3D, fatostatin at higher concentrations (10 and 20 μM) significantly increased caspase-3/7 activity in PC-3 R248W and DU145 cells but not in PC-3 EV cells. Similar results were demonstrated by Western blot, where fatostatin led to increases of the cleavage of caspase-9, caspase-3 and PARP in both PC-3 R248W and DU145 cells (Figure 3E). Taken together, the results of cell cycle and apoptosis studies indicate that fatostatin causes G_2_/M cell cycle arrest and induces caspase-dependent apoptotic death in PCa cells carrying mutant p53s.

### Fatostatin significantly suppresses mutant p53-activated SREBP metabolic pathways in PCa cells

**Figure 4:**
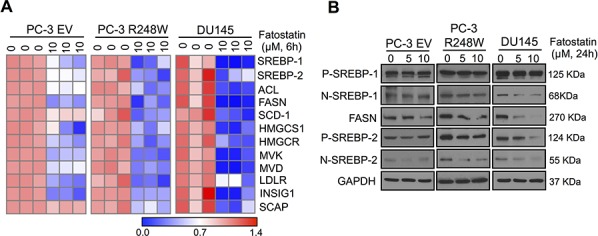
Fatostatin predominately suppresses mutant p53-activated SREBP signaling pathways in PCa cells **A.** Heat map showing qPCR analysis of SREBP-1, SREBP-2 and their downstream targets in PC-3 EV, PC-3 R248W and DU145 cells treated with fatostatin for 6 hours. The data were normalized by β-actin. **B.** Western blot analysis of SREBP-1, SREBP-2 and FASN expression in PC-3 EV, PC-3 R248W and DU145 cells treated with fatostatin for 24 hours. GAPDH was used as a loading control.

To reveal the molecular mechanism underlying the inhibitory effect of fatostatin in PCa cells harboring mutant p53s, we first examined the transcriptional expression of SREBPs and their anabolic genes [16] affected by fatostatin: ATP citrate lyase (ACL), FASN and stearoyl-CoA desaturade-1 (SCD-1) for lipogenesis; and 3-hydroxy-3-methyl-glutaryl-CoA synthase 1 (HMGCS1), HMGCR, mevalonate kinase (MVK), mevalonate 5-pyrophosphate decarboxylase (MVD) and low-density lipoprotein receptor (LDLR) for cholesterogenesis; and two chaperones, insulin-induced gene 1 (INSIG1) and SREBP cleavage activating protein (SCAP) in PC-3 EV, PC-3 R248W and DU145 cells. Fatostatin significantly down-regulated mRNA expression of these genes in all tested cells, with more effective reduction detected in PC-3 R248W and DU145 cells carrying mutant p53s, (Figure 4A). Similar results were observed by Western blot analysis, where fatostatin reduced expression of SREBP-1, SREBP-2 and FASN proteins (Figure 4B). These results indicate that fatostatin predominantly inhibited the SREBP metabolic pathways in PCa cells harboring mutant p53s.

Reactivation of wild-type function in mutant p53 protein has been a major strategy for treating human cancer harboring mutant p53s such as small molecules, STIMA-1 [27] and APR-246 [28]. To determine the restoration of wtp53 by fatostatin, we performed qPCR analysis of wtp53 target genes [29, 30], including p21, p53R2, BAX and MDM2 in PC-3 R248W and DU145 cells. Fatostatin did not affect mRNA expression of these target genes in PC-3 R248W and DU145 cells (Supplementary Figure 3A). Western blot analysis also showed that fatostatin had no effect on p21^Waf1/Cip1^ expression in PC-3 R248W and DU145 cells (Supplementary Figure 3B). Our data suggest that fatostatin did not restore wtp53 activity in PCa cells carrying mutant p53s. Taken together, these results indicate that fatostatin predominantly inhibited the SREBP metabolic pathways mediated by mutant p53s in PCa cells with p53 mutations.

### Fatostatin synergizes with docetaxel to inhibit cell proliferation and induce apoptosis in PCa cells harboring mutant p53s

Docetaxel is the first-line chemotherapy to improve survival in mCRPC patients after the failure of hormone therapy [7]. However, the resistance to docetaxel rapidly develops and is closely associated with p53 mutation status [8]. As shown in Supplementary Figure 4A, the IC_50_ value of docetaxel in PC-3 R248W cells was 217.9 nM, which was higher than that in PC-3 EV cells (24.8 nM), suggesting that mutant p53 could be a crucial determinant for docetaxel resistance in PCa cells. Moreover, docetaxel treatment led to the activation of SREBP expression in DU145 cells (Supplementary Figure 4B). Therefore, we hypothesize that docetaxel-based combination therapy targeting SREBP-mediated pathways may delay the onset of docetaxel resistance and provide a new potential strategy for the treatment of mCRPC harboring mutant p53s.

**Figure 5:**
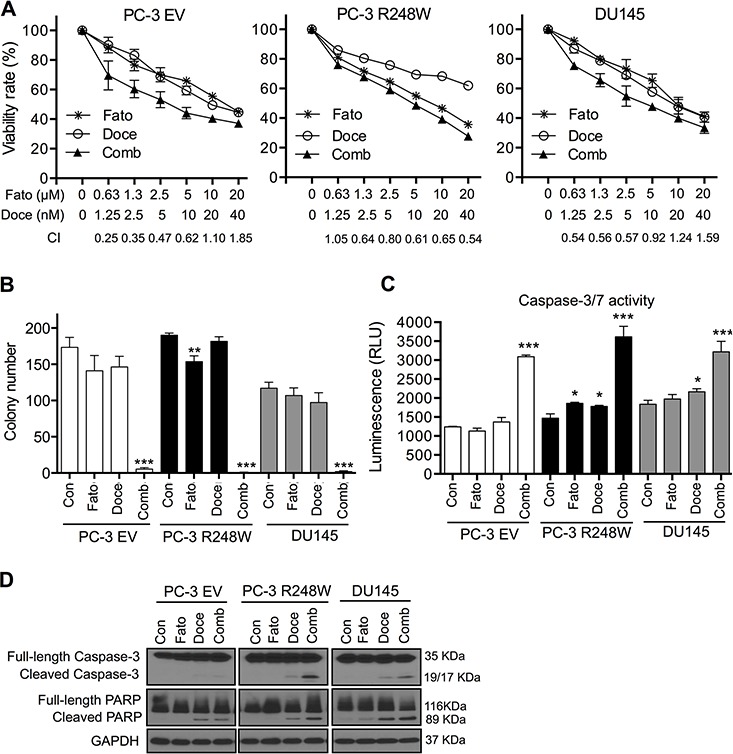
Fatostatin synergizes with docetaxel to inhibit cell proliferation and induce apoptosis in PCa cells harboring different p53 status **A.** The combination of fatostatin and docetaxel suppressed cell viability in PC-3 EV, PC-3 R248W and DU145 cells. Fato, fatostatin; Doce, docetaxel; Comb, combination. The combination index (CI) was calculated by Calcusyn software and is shown at the bottom. **B.** The combination of fatostatin (2.5 μM) and docetaxel (5.0 nM) significantly decreased colony formation by PC-3 EV, PC-3 R248W and DU145 cells. The number of colonies was counted and reported as the mean ± SD of triplicate experiments. **P < 0.01, ***P < 0.001. Con, control. **C.** The combination treatment significantly increased caspase-3/7 activity of PC-3 EV, PC-3 R248W and DU145 cells. Data are plotted as the relative units of luciferase intensity and reported as the means ± SD values from triplicate experiments. **P < 0.01, ***P < 0.001. **D.** Western blot analysis of apoptosis-related markers (caspase-3 and PARP) in PC-3 EV, PC-3 R248W and DU145 cells treated with fatostatin, docetaxel, alone or in combination for 72 hours. GAPDH was used as a loading control.

To test this idea, we first investigated the effect of fatostatin in combination with docetaxel on the growth of PC-3 EV, PC-3 R248W and DU145 cells. Cells were treated with sequential doses of fatostatin and docetaxel, alone or in combination, for 72 hours and the combination index (CI) values were subsequently calculated using the Calcusyn software [31]. As shown in Figure 5A, the combination of fatostatin with docetaxel exhibited significant synergistic inhibitory effects on the growth of these cells. The CI analysis showed statistically significant synergy with Log (CI) <0, especially in PC-3 R248W cells (Supplementary Figure 4C). To further investigate the combinational effect of these two drugs, cells were treated with fatostatin and docetaxel alone or in combination and cell growth was determined. As shown in Supplementary Figure 4D, the combination treatment was more effective in inhibiting cell growth than single agent treatment. In concordance with growth inhibition, the combination treatment displayed significantly reduced clonogenic capacity of the tested cell lines compared with single agent treatment at a low concentration of fatostatin (2.5 μM) or docetaxel (5.0 nM) (Figure 5B and Supplementary Figure 4E). These data suggest that the combination of fatostatin and docetaxel resulted in significant inhibition of cell growth and colony formation in metastatic PCa cells with null and/or mutant p53s.

Given the synergistic growth inhibitory effect of fatostatin and docetaxel, we next determined the impact of the combination on apoptosis. PC-3 EV, PC-3 R248W and DU145 cells were treated with fatostatin and/or docetaxel for 72 hours and subjected to caspase-3/7 activity analysis. Treatment with fatostatin alone at the concentration of 2.5 μM resulted in an increase of caspase-3/7 activity in PC-3 R248W cells compared with vehicle treatment, which was not observed in PC-3 EV and DU145 cells. However, a significant increase of caspase-3/7 activity was observed in all cell lines in the presence of both fatostatin and docetaxel (Figure 5C). Additionally, Western blot analysis demonstrated that the combination of fatostatin and docetaxel significantly increased expression of cleaved caspase-3 and cleaved PARP proteins in PC-3 R248W and DU145 cells, but resulted in a small increase of cleaved PARP in PC-3 EV cells compared with fatostain or docetaxel alone, respectively (Figure 5D). Collectively, these data suggest that the combination of fatostatin and docetaxel has significant synergistic effects on the induction of apoptosis in PCa cells, especially those with mutant p53s.

### Fatostain alone or in combination with docetaxel inhibits PCa tumor growth and blocks SREBP-regulated pathways in subcutaneous xenograft mouse models

**Figure 6:**
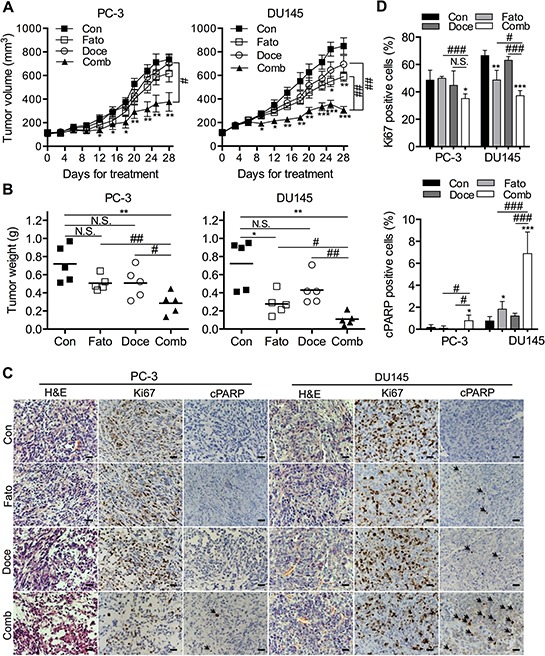
Fatostain alone or in combination with docetaxel inhibits PCa tumor growth in a subcutaneous xenograft mouse model **A.** PC-3 and DU145 cells were subcutaneously injected in the flanks of male athymic mice. Tumor volume was monitored for 4 weeks after treatment with fatostatin and/or docetaxel. Each point represents the mean ± SEM of the measured tumor volume (*N* = 5/group). **P < 0.01, ***P < 0.001, compared with the control group; #*P* < 0.05, ##*P* < 0.01, compared with the combination group. Con, control; Fato, fatostatin; Doce, docetaxel; Comb, combination. **B.** Subcutaneous PC-3 and DU145 tumors were weighed. N.S., no significance; *P < 0.05, **P < 0.01, compared with the control group; #*P* < 0.05, ##*P* < 0.01, compared with the combination group. **C.** Representative H&E and IHC staining of Ki67 and cleaved PARP (cPARP; black arrow) in PC-3 and DU145 tumor sections collected from control, fatostatin-, docetaxel- and combination-treated groups. Cell nuclei were counterstained with hematoxylin (blue). Scale bar = 50 μm. **D.** Quantitative analysis of Ki67 and cPARP was performed and reported as the percentage of Ki67 or cPARP positive cells in PCa xenograft tumors. N.S., no significance; *P < 0.05, **P < 0.01, ***P < 0.001, compared with the control group; #*P* < 0.05, ###*P* < 0.001, compared with the combination group.

In view of the potent *in vitro* effects of fatostatin and docetaxel on the growth of p53 null or mutant PCa cells (parental PC-3 and DU145 cells), we next evaluated the therapeutic potential of fatostatin and/or docetaxel in immunodeficient mice. The *in vivo* results showed that fatostatin effectively reduced tumor size (Figure 6A, right panel) and tumor weight (Figure 6B, right panel) of DU145 xenografts compared to an untreated control group, whereas fatostatin alone did not significantly affect the growth of PC-3 xenograft tumors (Figures 6A and 6B, left panel). The combination of fatostatin and docetaxel exhibited greater inhibitory effect on both PC-3 and DU145 xenograft models without showing significant toxicity (body weight) compared to individual drugs (Figures 6A and 6B; Supplementary Figures 5A and 5B). In addition, the expression of proliferation (Ki67) and apoptosis (cPARP) markers in PC-3 and DU145 tumor specimens was examined by IHC staining. Fatostatin treatment decreased expression of Ki67 and increased expression of cPARP in DU145 xenograft tumors compared to the vehicle-treated group, while no obvious changes in PC-3 xenograft tumors between the vehicle and fatostatin-treated groups were shown (Figures 6C and 6D). The combination of fatostatin and docetaxel led to a reduction of Ki67 expression and induction of cPARP expression in both PC-3 and DU145 xenograft tumors (Figures 6C and 6D). Collectively, the *in vivo* data suggest that the combination of fatostatin and docetaxel has significant anti-tumor efficacy in PCa xenograft models.

**Figure 7:**
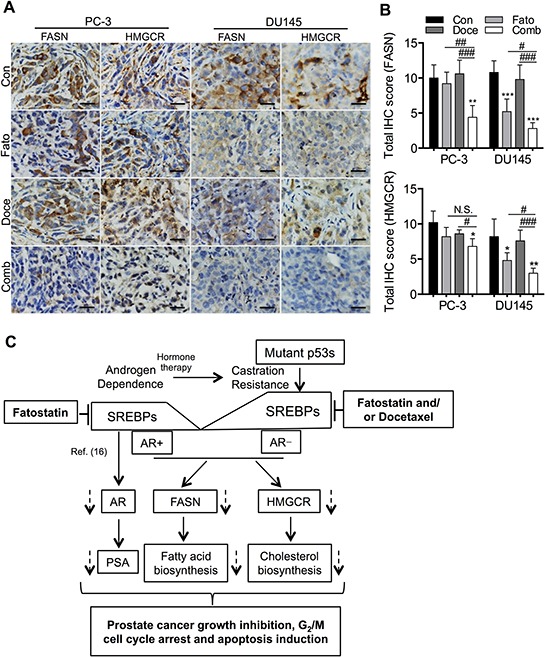
Fatostain alone or in combination with docetaxel blocks the SREBP-regulated pathways in a PCa subcutaneous xenograft mouse model **A.** Expression of FASN and HMGCR was examined by IHC staining in subcutaneous PC-3 and DU145 tumor sections collected from control, fatostatin-, docetaxel- and combination-treated groups. Scale bar = 50 μm. **B.** Quantitative analysis of FASN and HMGCR was performed and reported as total IHC score in PCa xenograft tumors. N.S., no significance; *P < 0.05, **P < 0.01, ***P < 0.001, compared with the control group; #*P* < 0.05, ###*P* < 0.001, compared with the combination group. **C.** A proposed model of the mechanism underlying growth inhibition, G_2_/M cell cycle arrest and apoptosis induction by fatostatin and/or docetaxel in AR-negative PCa harboring p53 mutations or by fatostatin alone in AR-positive PCa (Ref. 16) through blockade of the SREBP-mediated pathways.

We subsequently evaluated the expression of SREBP downstream target genes, FASN and HMGCR, two key enzymes for controlling the rate-limiting step of lipogenesis and cholesterogenesis in PC-3 and DU145 xenograft tumors treated with fatostatin and/or docetaxel by IHC staining. Consistent with the *in vitro* data, fatostatin significantly decreased expression of FASN and HMGCR in DU145 xenograft tumors but not in PC-3 xenograft tumors compared with the respective vehicle group (Figures 7A and 7B). In addition, the combination of fatostatin and docetaxel treatment led to a reduction in expression of FASN and HMGCR in both PC-3 and DU145 xenograft tumors (Figures 7A and 7B). The *in vivo* results collectively indicate that efficient anti-tumor activity resulting from the combination of fatostatin and docetaxel treatment is associated with deregulation of the SREBP-mediated pathways in PCa, especially those with p53 mutations.

## DISCUSSION

Mutant p53s have been well demonstrated to promote cancer progression and are implicated in survival, invasion/migration and metastasis through a series of molecular mechanisms, including alternations in DNA-binding ability, changes in the interaction with other functional proteins or indirectly involvement in transcriptional regulation [25, 32]. Additionally, PCa resistance to docetaxel treatment, which is currently considered incurable, has been shown to be correlated with p53 mutations [8]. In the present study, we provide new evidence that mutant p53s regulate expression of SREBPs and their downstream genes, which have been shown to affect multiple aspects of PCa, including tumorigenesis, castration resistance and metastasis [13, 14]. This novel discovery provides a rationale for targeting the mutant p53s/SREBP-mediated pathways as a promising approach to treat aggressive human cancer harboring mutant p53s and prevent lethal docetaxel-resistant progression.

Emerging evidence suggests that wtp53/mutant p53 is involved in regulating lipid metabolism, and this alteration may contribute to disease progression in breast cancer [9, 10]. Our results support the idea that overexpression of tumor suppressor wtp53 inhibited expression of SREBP-1 and -2 as well as their targets associated with the rate-limiting steps of lipogenesis and cholesterogenesis, FASN and HMGCR, in PC-3 cells. Moreover, mutant p53s induced activation of SREBP-1 and -2, leading to increased expression of FASN and HMGCR in PC-3 cells. Conversely, knockdown of endogenous mutant p53s resulted in inhibition of SREBPs, FASN and HMGCR expression. Collectively, these mechanistic data indicated that mutant p53s play an important role in the activation of the SREBP-regulated metabolic pathways in PCa cells.

We and others previously reported that targeting SREBPs transcriptional activities and their downstream metabolic pathways was a promising therapeutic approach for the treatment of PCa [15, 16, 33, 34]. Fatostatin, a new non-sterol synthetic diarylthiazole derivative, has been shown to inhibit insulin-induced adipogenesis in mouse fibroblast cells and decrease blood fatty acid and cholesterol and body weight in obese mice [17]. We demonstrated that fatostatin displayed anti-cancer activity by interrupting SREBP transcriptional activity in AR-positive PCa cells [16]. AR is a critical growth and survival factor in regulation of PCa development and aggressiveness. We identified an active SREBP binding site in the 5′-flanking AR promoter region [35]. Through inhibition of SREBP transcriptional activity, fatostatin reduced the expression of AR gene and its downstream target, prostate-specific antigen (PSA), in LNCaP and C4-2B cells [16]. PCa is a heterogeneous and biologically complex disease harboring genomic alterations, involving the AR and p53 genes, and exhibiting aggressive features. The efficacy of fatostatin on metastatic AR-negative PCa cells with null or mutant p53s is unknown. In this study, we revealed that blocking the SREBP-regulated metabolic pathways by fatostatin had a better inhibitory on the proliferation of PCa cells with high levels of mutant p53 (PC-3 R248W and DU145) than cells with null (PC-3 EV) or low levels of mutant p53 (DU145 shp53). Moreover, pharmacological inhibition of SREBP by fatostatin significantly suppressed xenograft DU145 tumor growth in immunodeficient nude mice by decreasing Ki67 expression and increasing cPARP expression. These in vitro and in vivo results and an earlier report [16] collectively suggest that fatostatin not only exhibits anti-tumor activity in AR-positive PCa cells but also shows efficacy on metastatic AR-negative PCa with p53 mutations.

Uncontrolled cellular division and growth are cancer hallmarks. Thus, cell cycle blockade has been considered as an attractive therapeutic strategy to eliminate cancer [36, 37]. Eukaryotic cell cycle progression is regulated by the coordinated activity of cyclin-dependent kinase (Cdk) and cyclin complexes [38]. The G_2_/M transition is largely dependent on Cyclin B1 and Cdk1 activity, which can be regulated by p21^Waf1/Cip1^ [39]. We found that decreased Cyclin B1 and p-Cdk1 expression was accompanied by G_2_/M transition blockade in fatostatin-treated PC-3 R248W and DU145 cells. Additionally, fatostatin had no effect on the expression of wtp53 downstream targets such as p21^Waf1/Cip1^ in PCa cells carrying mutant p53s. These data suggest that fatostatin induces DNA damage signaling in a p53 independent manner to trigger cell cycle arrest in the G_2_ phase in PC-3 cells harboring mutant p53s. The intrinsic (mitochondrial-mediated) and extrinsic (death receptor-mediated) apoptotic pathways converge on the same terminal “execution” pathway, which is initiated by the cleavage of caspases [40]. Upon activation of caspase-3, substrates such as PARP are cleaved, ultimately leading to programmed cell death [41]. Treatment with fatostatin induced the activation of mitochondrial-related caspase-9, and -3 and the cleavage of PARP but not caspase-8 (data not shown) in PC-3 R248W and DU145 cells, which reveal that fatostatin induces programmed cell death in PCa cells harboring mutant p53s mainly via an intrinsic mitochondrial-dependent apoptotic pathway.

Docetaxel is a standard treatment for mCRPC patients. In response to docetaxel, most PCa cells harboring mutant p53s show reduced sensitivity compared to cells lacking p53 or those with wtp53 [8]. Consistent with our observation, higher IC_50_ of docetaxel was detected in PC-3 R248W cells than that in PC-3 EV cells. Importantly, docetaxel treatment increased expression of SREBP-1 and -2 in DU145 cells. Based on these findings, co-targeting p53 mutation and its associated pathways activated by docetaxel may improve the therapeutic effectiveness of current anti-PCa drugs and delay docetaxel resistance in PCa. In the current study, the combination of fatostatin with docetaxel displayed synergistic growth inhibition and apoptosis induction in PCa cells harboring null or mutant p53s *in vitro* and *in vivo*. Notably, this combination was strongly synergistic in p53 mutant PCa cells, which suggests that this novel therapeutic approach might exhibit an improved selective advantage in metastatic PCa cells harboring mutant p53s. Moreover, the docetaxel has been shown to lead to CRPC tumor regression via impairing AR nuclear translocation and activity by a microtubule-associated mechanism [42]. Fatostatin inhibited the expression of AR and PSA genes through interrupting SREBP transcriptional activity [16]. These promising mechanistic findings provide a critical insight into the treatment of advanced PCa aggressiveness, such as mCRPC, through the concerted targeting of multiple pathways by the combination of fatostatin and docetaxel (Figure 7C). Currently, docetaxel-resistant PCa cells are establishing. These docetaxel-resistant PCa cells as well as other cancer cell lines, such as breast, lung and renal cancer cells, will be applied to investigate the therapeutic benefit of fatostatin and/or docetaxel in the near future.

In summary, we provide evidence that targeting the SREBP-regulated metabolic pathways by fatostatin inhibited cell proliferation and induced apoptosis in metastatic PCa cells harboring p53 mutations. Additionally, we revealed an innovative preclinical concept by combining fatostatin with docetaxel to achieve a strong synergistic anti-cancer effect on PCa cell lines and xenograft mouse models. Our findings suggest that fatostatin alone or in combination with docetaxel can be a potent therapeutic strategy for the treatment of aggressive PCa, especially PCa harboring mutant p53s.

## MATERALS AND METHODS

### Cell cultures and reagents

Human metastatic prostate cancer PC-3 (p53-null) and DU145 (p53-mutant) cell lines were obtained from American Type Culture Collection (ATCC) and grown in RPMI-1640 medium supplemented with 10% fetal bovine serum (Atlanta biological, Flowery Branch, GA), penicillin (100 U/ml) and streptomycin (100 μg/ml) at 37°C in a humidified incubator with 5% CO_2_. Human wtp53, various p53 mutants (V143A, R248W, R175H and R273H) and control expression constructs were obtained from Addgene (Cambridge, MA). Human p53 shRNA lentiviral particles were purchased from Santa Cruz Biotechnology (Santa Cruz, CA). Fatostatin and docetaxel were purchased from Chembridge Corporation (San Diego, CA) and Selleck Chemicals (Houston, TX), respectively.

### Plasmids transfection and viral transduction

For transfection experiments, PC-3 cells were transfected with wtp53, various p53 mutants or empty vectors using Lipofectamine LTX Plus reagent (Life Technologies, Carlsbad, CA), following the manufacturer's instructions. Stable cell clones were established after the selection with 600 μg/ml G418 (Sigma-Aldrich, St. Louise, MO). For p53 knockdown studies, p53-targeting shRNA or scrambled shRNA lentiviral particles were used to infect DU145 cells followed by puromycin (Life Technologies) selection to obtain stable cell clones.

### Western blot analysis

Cells were harvested and subjected to Western blot analysis as described previously [14]. The primary antibodies used in the experiments were as follows: p53, Cyclin B1, Cdk1, phospho-Cdk1 (p-Cdk1 Tyr15), capases-9, poly (ADP-ribose) polymerase (PARP), GAPDH (Cell Signaling Technology, Danvers, MA), caspase-3 (Novus Biologicals, Littleton, CO), SREBP-1, FASN, HMGCR (Santa Cruz Biotechnology), and SREBP-2 (Abcam, Cambridge, MA).

### Quantitative reverse transcription-polymerase chain reaction

qPCR analysis was performed as previously described [15]. Total RNA from cells was isolated by RNeasy Mini Kit (Qiagen, Valencia, CA) and converted into cDNA using an iScript cDNA Synthesis Kit (Bio-Rad, Hercules, CA). Quantitative polymerase chain reaction (qPCR) was performed using the SYBR Green PCR Master Mix on an ABI 7500 Fast Real-Time PCR System (Applied Biosystems, Grand Island, NY) Sequences of primers for TP53, SREBP-1, SREBP-2, ACL, FASN, SCD-1, HMGCS1, HMGCR, MVK, MVD, LDLR, INSIG1, SCAP, P21, P53R2, BAX, MDM2 and β-actin are summarized in Supplemental Table 1.

### Cell proliferation and clonogenic assays

Cell proliferation and clonogenic assays were conducted as previously described [15, 16]. To determine the potential mechanism of drug-drug interactions, we used CompuSyn software (Cambridge, UK) to calculate the combination index (CI). A CI of less than 1.0 was considered to be indicative of synergism.

### Cell cycle and apoptosis analysis

Cell cycle distribution, apoptotic cell death and caspase activity assays were performed as previously described [15, 16].

### Xenograft tumor mouse model

All animal procedures were performed in accordance with a protocol approved by the Institution Animal Care and Use Committee at Cedars-Sinai Medical Center. The *in vivo* efficacy of fatostatin and/or docetaxel was investigated by subcutaneously implanting parental PC-3 or DU145 cells (1 × 10^6^) in the flank of 4-week-old male athymic nude mice (Harlan Laboratories, Indianapolis, IN) in a PBS and BD Matrigel matrix mixture (100 μl; 1:1, v/v). Mice bearing PC-3 or DU145 tumors with a mean volume of 100 mm^3^ were randomly distributed into vehicle control (sterile PBS), fatostatin (15 mg/kg), docetaxel (5 mg/kg) or combination treated groups (five mice each group). Mice were weighted once a week and administered by intraperitoneal (i.p.) injection once daily for fatostatin and twice a week for docetaxel [16, 17]. During 4-week treatment, tumor volumes were measured by the caliper and calculated using the formula: *V* = 1/2 × length × width^2^.

### Immunohistochemical Staining

Immunohistochemical (IHC) staining was performed on xenograft tumor tissues fixed with 4% paraformaldehyde, paraffin-embedded, and sectioned as described previously [35]. Antibodies used for IHC staining included Ki67 (Abcam), cleaved PARP, FASN and HMGCR. The percentage of Ki67 positive cells (proliferation) or cleaved PARP (cPARP) positive cells (apoptosis) [16] in tumor tissues from all groups were calculated in 5 randomly selected microscopic fields at a 200 × magnification. The total IHC score for FASN and HMGCR staining was calculated as the value of the percentage of positive staining × staining intensity, and ranged from 0 to 12 [43, 44].

### Statistical analysis

The quantitative data are presented as the means ± SD (SEM). Statistically significant differences were determined by an unpaired Student's *t* test. A value of *P* < 0.05 was considered to be statistically significant.

## SUPPLEMENTARY FIGURE AND TABLE


