# Supplementary material for: Anti-cancer efficacy of SREBP inhibitor, alone or in combination with docetaxel, in prostate cancer harboring p53 mutations
Source: Oncotarget. 2015 Oct 16;6(38):41018–32. doi: 10.18632/oncotarget.5879 (PMC4747386; doi:10.18632/oncotarget.5879)
Supplement: Supplementary file 1 [file oncotarget-06-41018-s001.pdf]

SUPPLEMENTARY FIGURE AND TABLE

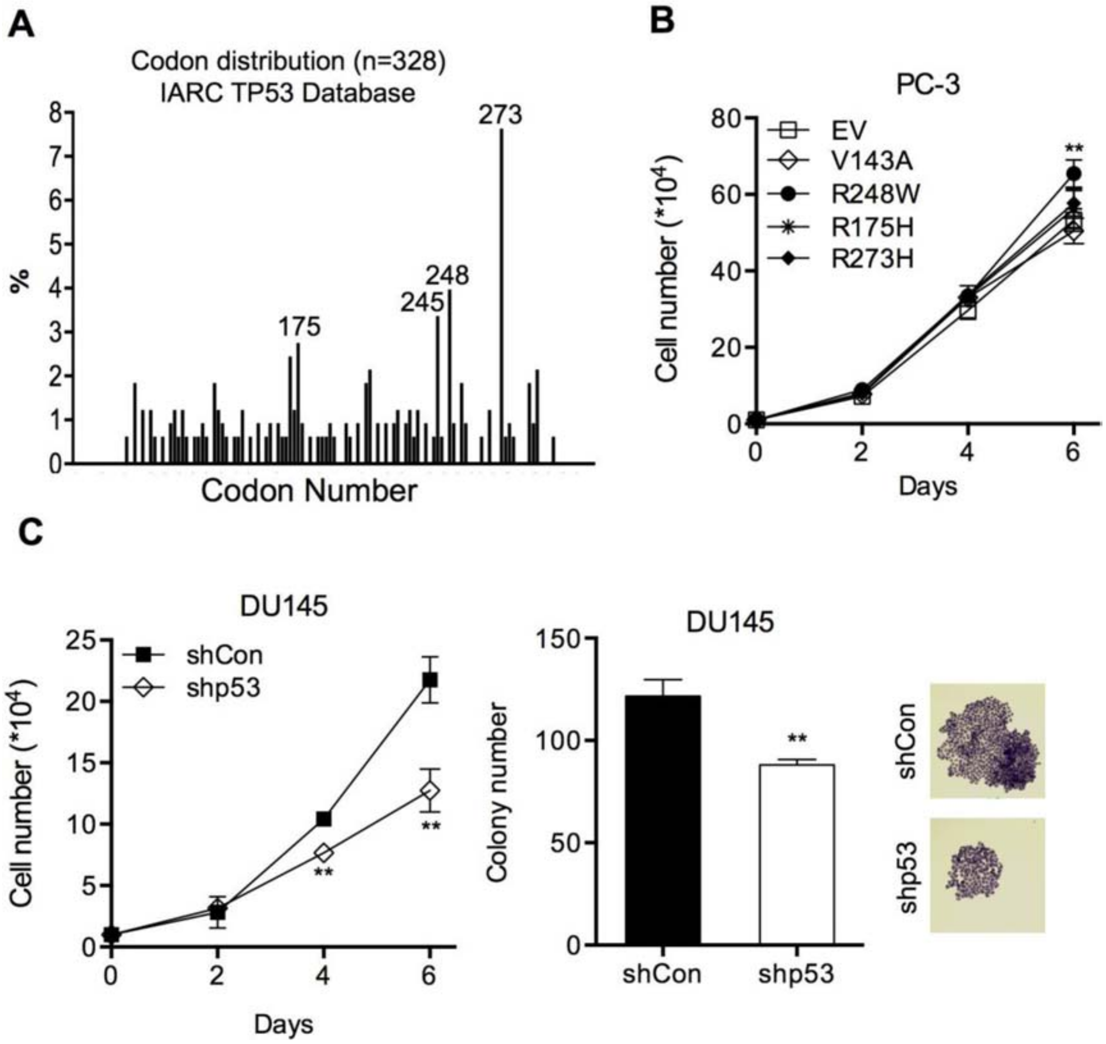

Supplementary Figure S1: The effects of mutant p53 on cell growth and colony formation in PCa cells. A. Distribution of human p53 gene mutations in PCa. **B.** Growth curve of PC-3 cells stably transfected with empty vector or various mutp53s. $^{**}P < 0.01$. **C.** Growth curve (left panel) and colony formation (right panel) of DU145 cells infected with shp53 or shCon letivirus particles. $^{**}P < 0.01$.

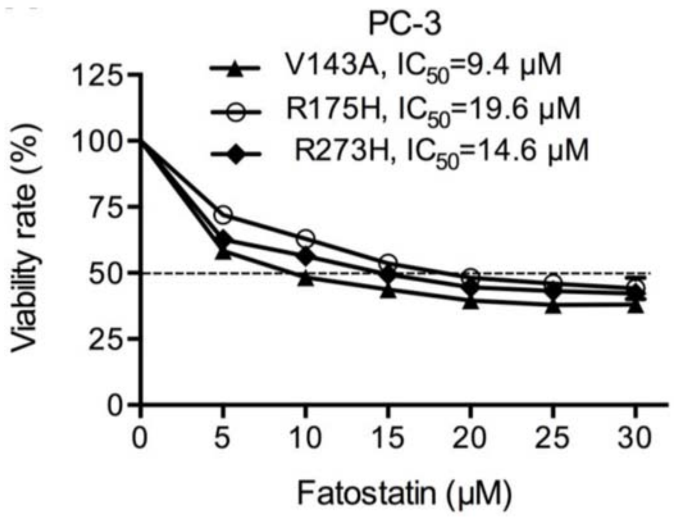

Supplementary Figure S2: Fatostatin inhibited cell proliferation of PCa cells harboring p53 mutations. PC-3 V143A, R175H and R273H cells were treated with various concentrations of fatostatin and incubated for 72 hours. The IC_{50} value of each cell line was calculated and shown from three independent experiments.

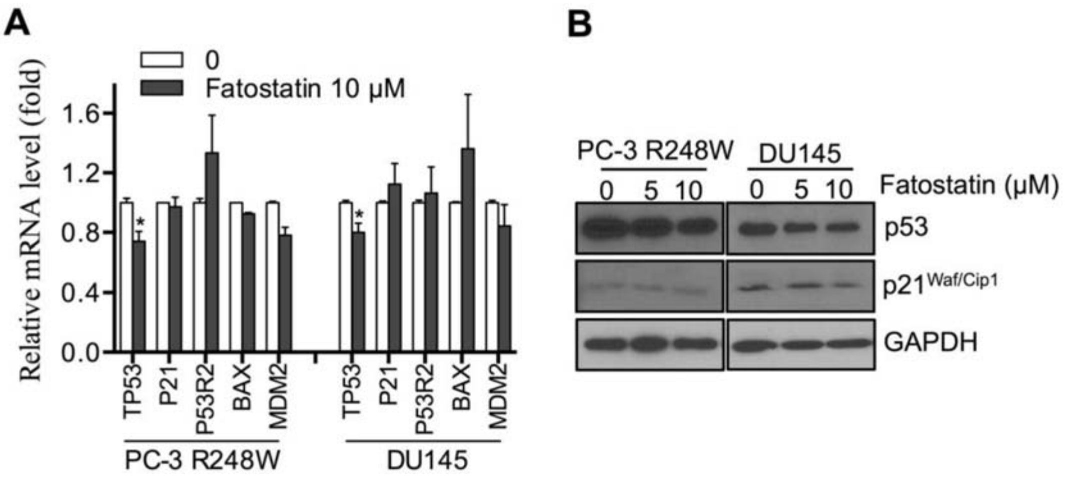

Supplementary Figure S3: The effect of fatostatin on wild-type p53 downstream targets in PC-3 R248W and DU145 cells. A. qPCR analysis of a series of wild-type p53 targets, including P21, P53R2, BAX and MDM2 in PC-3 R248W and DU145 cells treated with fatostatin for 6 hours. *$P < 0.05$. **B.** Western blot analysis of p53 and $p21^{Waf/Clip1}$ expression in PC-3 R248W and DU145 cells treated with fatostatin for 24 hours.

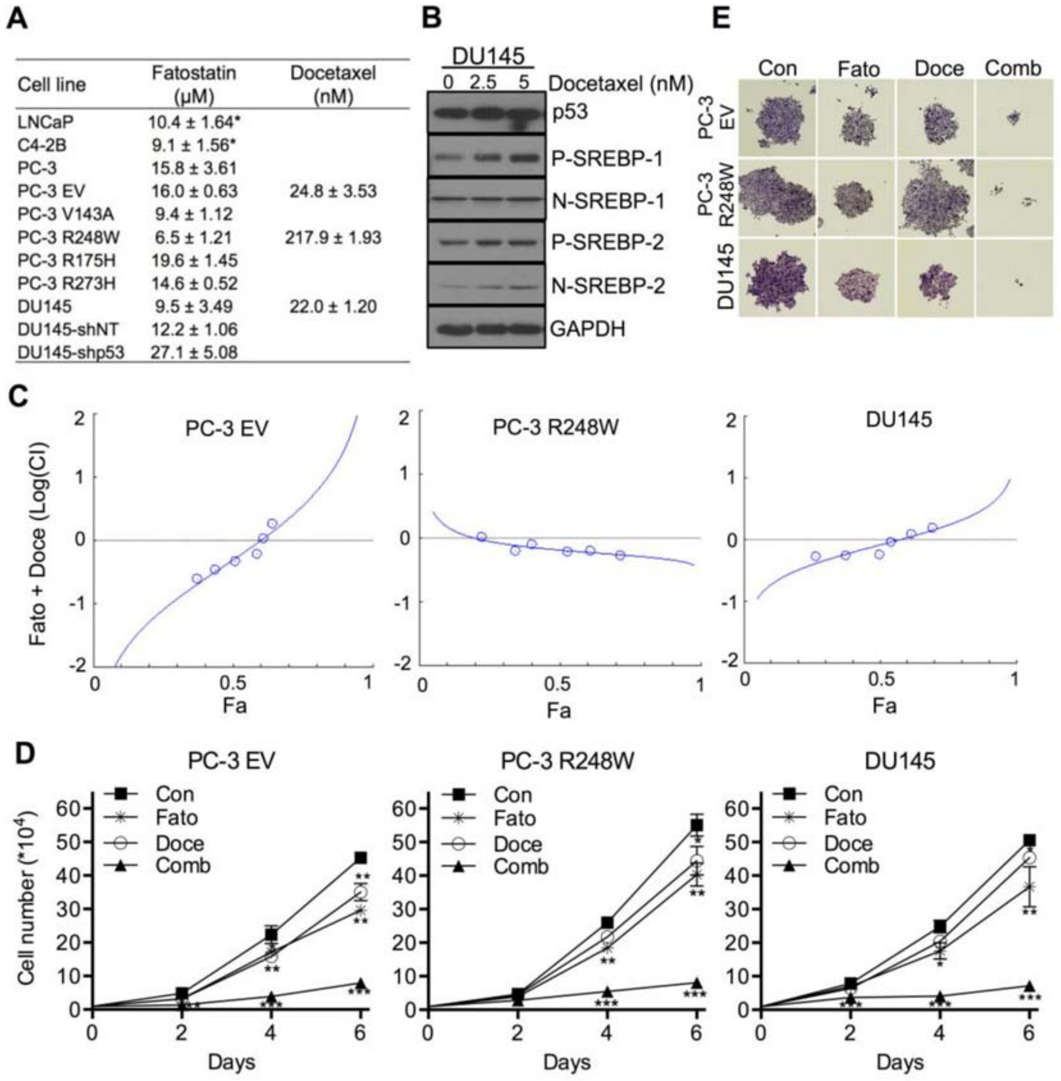

Cell line	Fatostatin (µM)	Docetaxel (nM)
LNCaP	10.4 ± 1.64*	
C4-2B	9.1 ± 1.56*	
PC-3	15.8 ± 3.61	
PC-3 EV	16.0 ± 0.63	24.8 ± 3.53
PC-3 V143A	9.4 ± 1.12	
PC-3 R248W	6.5 ± 1.21	217.9 ± 1.93
PC-3 R175H	19.6 ± 1.45	
PC-3 R273H	14.6 ± 0.52	
DU145	9.5 ± 3.49	22.0 ± 1.20
DU145-shNT	12.2 ± 1.06	
DU145-shp53	27.1 ± 5.08	

Supplementary Figure S4: The combination of fatostatin with docetaxel inhibited cell proliferation and colony formation in PCa cells harboring different p53 mutations. **A.** The inhibitory effects of fatostatin and docetaxel on PCa cells. IC_{50} was calculated and shown as the mean ± SD of three independent experiments. *, previously published data. **B.** Western blot analysis of p53, SREBP-1 and SREBP-2 expression in DU145 cells treated with docetaxel for 24 hours. *P* and *N* denote the precursor and nuclear forms of SREBP, respectively. **C.** The combination index (CI) values of fatostatin and docetaxel in PC-3 EV, PC-3 R248W and DU145 cells were calculated using Calcusyn software and reported as Log (CI) for each fraction affected (Fa). Fato, fatostatin; Doce, docetaxel. **D.** Cell growth of PC-3 EV, PC-3 R248W and DU145 cells treated with fatostatin (2.5 µM), docetaxel (5.0 nM), alone or in combination. *$P < 0.05$, **$P < 0.01$, ***$P < 0.001$ compared with control. Con, control; Comb, combination. **E.** Images for colonies of PC-3 Vector, PC-3 R248W and DU145 cells treated with fatostatin and docetaxel, alone or in combination.

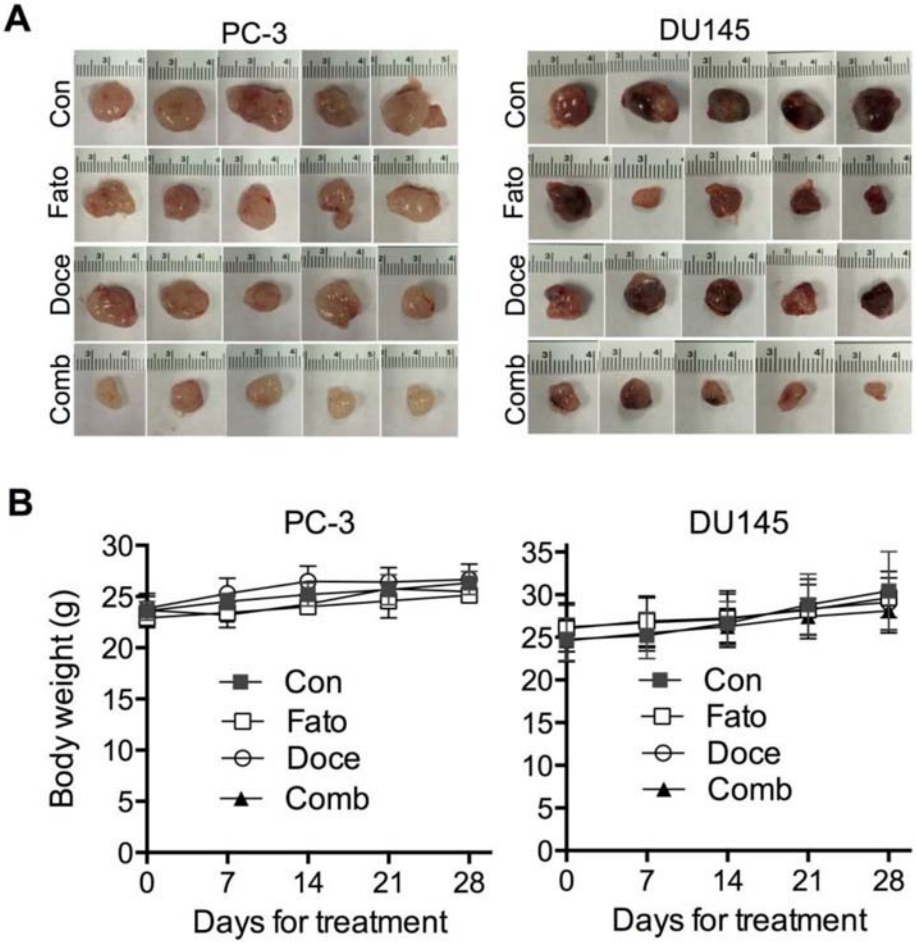

Supplementary Figure S5: The combination of fatostatin with docetaxel inhibited PCa tumor growth in xenograft models. A. Representative tumor images derived from PC-3 and DU145 tumor xenograft models treated with vehicle, fatostatin and/or docetaxel. **B.** Body weight of mice bearing PC-3 or DU145 tumors treated with vehicle, fatostatin and/or docetaxel. Con, Control; Fato, fatostatin; Doce, docetaxel; Comb, combination.

Supplementary Table S1. The sequences of primers used for qPCR

Gene	Forward (5′-3′)	Reverse (5′-3′)
TP53	CCCAAGCAATGGATGATTTGA	GGCATTCTGGGAGCTTCATCT
SREBP-1	TCAGCGAGGCGGCTTTGGAGCAG	CATGTCTTCGATGTCGGTCAG
ACL	TGTAACAGACCAGGAACCC	CTGTACCCCAGTGGCTGTTT
FASN	CGGTACGCGACGGCTGCCTG	GCTGCTCCACGAACTCAAACACCG
SCD-1	CACTTGGGAGCCCTGTATGG	AGCCGAGCTTTGTAAGAGCG
SREBP-2	CCCCTGACTTCCCTGCTGCA	GCGCGAGTGTGGCCGGATC
HMGCS1	GAGGGCTTCGTGGGACACATA	GCCACTGGGATGGATCTTT
HMGCR	GTCATTCCAGCCAAGGTTGT	GGGACCACTTGCTTCCATTA
MVK	CCTTGTGGCTGGCGTCAGAAA	CGAGGGCATTCAGATGGTGCT
MVD	ACCACGGGGACACCACGGT	CCACACAGCAGCCACAAACTC
LDLR	CAACGGCTCAGACGAGCAAG	AGTCACAGACGAACTGCCGAGA
INSIG1	GGACGACAGTTAGCTATGGGTGTT	GAGTCATTTGTACAGTCAGCCCGA
SCAP	TATCTCGGGCCTTCTACAACCA	ACACAACTCCTCCAAGCTCCTG
P21	GGCAGACCAGCATGACAGATT	GCGGATTAGGGCTTCCTCT
P53R2	GAAAGGGTCAGGGAGATCAT	TTCTGCCTGAAAAACCTTTG
BAX	TGG AGCTGCAGAGGATGATTG	GAAGTTGCCGTCAGAAAACATG
MDM2	GCAAATGTGCAATACCAACATGTC	GCCAAACAAATCTCCTAGAAGATC
β-actin	CAAGGCCAACCGCGAGAAGATGAC	GCCAGAGGCGTACAGGGATAGCACA
